# Morphine and microRNA Activity: Is There a Relation with Addiction?

**DOI:** 10.3389/fgene.2012.00223

**Published:** 2012-11-09

**Authors:** Raquel E. Rodríguez

**Affiliations:** ^1^Department of Biochemistry and Molecular Biology, Institute of Neuroscience, University of SalamancaSalamanca, Spain

**Keywords:** opioid, morphine, addiction, miRNA, zebrafish, dopaminergic system, miR-133b

## Abstract

When we talk about drug addiction, we are really dealing with an extremely complex system in which there still remain many unknowns and where many empty spaces or missing links are still present. Recent studies have identified changes in the expression profiles of several specific miRNAs which affect the interactions between these molecules and their targets in various illnesses, including addiction, and which may serve as valuable targets for more efficient therapies. In this review, we summarize results which clearly demonstrate that several morphine-related miRNAs have roles in the mechanisms that define addiction. In this regard, morphine has been shown to have an important role in the regulation of different miRNAs, such as miR-let-7 [which works as a mediator of the movement of the mu opioid receptor (MOR) mRNA into P-bodies, leading to translational repression], miR-23b (involved in linking MOR expression and morphine treatment at the post-transcriptional level), and miR-190 (a key post-transcriptional repressor of neurogenic differentiation, NeuroD). Fentanyl increases NeuroD levels by reducing the amount of miR-190, but morphine does not affect the levels of NeuroD. We also discuss the relationship between morphine, miRNAs, and the immune system, based on the discovery that morphine treatment of monocytes led to a decrease in several anti-HIV miRNAs (mir-28, 125b, 150, and 382). This review is centered on miR-133b and its possible involvement in addiction through the effects of morphine. We establish the importance of miR-133b as a regulatory factor by summarizing its activity in different pathological processes, especially cancer. Using the zebrafish as a research model, we discuss the relationship between mir-133b, the dopaminergic system, and morphine, considering: (1) that morphine modulates the expression of miR-133b and of its target transcript Pitx3, (2) the role of the zebrafish mu opioid receptor (zfMOR) in morphine-induced regulation of miR-133b, which depends on ERK1/2, (3) that morphine regulates miR-133b in hippocampal neurons, and (4) the role of delta opioid receptors in morphine-induced regulation of miR-133b. We conclude that the control of miR-133b levels may be a mechanism for the development of addiction to morphine, or other drugs of abuse that increase dopaminergic levels in the extracellular space. These results show that miR-133b is a possible new target for the design of new treatments against addictive disorders.

## Introduction

### Opioids

Opioids are the most potent compounds known today to control pain, and are also amongst the most used drugs of abuse (Corbett et al., 2006). They bind to the three classical opioid receptors, mu (MOR), delta (DOR), and kappa (KOR).

It has been established that the MOR displays higher affinity toward morphine than the other classical opioid receptors, delta, and kappa. Also, an interaction between the mu and the DOR has been described *in vitro*, which is thought to be responsible for faster development of morphine tolerance through the mu opioid receptor (Waldhoer et al., 2004). Moreover, when DOP are knocked out, mice do not develop tolerance to morphine and do not suffer the withdrawal effect once long-term treatment with morphine is over (Fundytus et al., 1995).

After establishing the pharmacological profiles of these classical opioid receptors, investigators studied post-transductional mechanisms, such as receptor internalization and desensitization. Later, interest focused on the intracellular effectors that mediate the activation of signaling cascades. It has been suggested that these post-transductional mechanisms are closely linked to the development of opiate tolerance and dependence on opiate drugs, mainly through the same plasticity mechanisms that produce adaptive changes in neural circuitries, for example, in memory and learning (Evans, 2004).

After more than 40 years of intense research on opiates, scientists now partly understand their mechanism of action on receptors.

Many issues concerning the mechanisms of addiction still remain to be established. This can be achieved by analyzing the different roles of the opioid receptors apart from their involvement in analgesic properties, such as their functions in developmental processes. For instance, the MOR and KOR increase neurogenesis (Kim et al., 2006), and the DOR acts as a neuroprotector (Narita et al., 2006). Also, other drugs of abuse, such as amphetamines, cocaine, or heroin, produce neuroadaptive changes in the brain that could be explained by shared gene regulatory mechanisms that lead to addiction. In this sense, opioid receptor gene regulation has been reported to be concurrently related to miRNAs and to the addiction process (Zheng et al., 2010b; Sanchez-Simon et al., 2010).

### Morphine

There are a wide variety of opiates, classified according to their origin and/or structure. The main natural opiates are morphine, phenanthrenic alkaloids similar to morphine (codeine and thebaine), and benzylisoquinolinic alkaloids (noscapine and papaverine). Semisynthetic opiates (heroin, hydrocodone, meperidine, oxycodone, buprenorphine, and etorphine) have a morphine-like structure, whereas fully synthetic opiates (methadone, fentanyl, tramadol, metazocine, and pentazocine) display a wide range of unrelated structures that show similar pharmacological properties. Unfortunately, opiates present some undesirable side effects, such as tolerance, dependence, and addiction (Nestler et al., 1993). Thus, there is a need for novel analgesic drugs that do not have these adverse side effects, especially in light of the widespread abuse of opiates.

The opioid alkaloide *Morphine* is the main active compound in opium, the juice obtained from the seed of the poppy plant *Papaver Somniferum*, and has been used for centuries as a medical and recreational agent. It is used both by medical patients suffering acute or chronic pain, and by habitual daily abusers. Since its isolation, morphine has been used largely for pain management, although other, non-analgesic uses, including experimental depression treatments and as a cure for opium addiction, have been developed. Despite these common uses, morphine produces disruptive negative secondary effects including sleepiness or drowsiness, blurred vision, constipation, and a decrease in blood pressure and appetite. With continuous use, morphine produces physical tolerance and addiction.

Accumulating evidence has demonstrated that, upon repeated exposure to morphine, long-lasting neurochemical alterations occur in discrete brain regions. Changes in gene expression are likely to mediate these adaptations in brain neurochemistry, thereby contributing to dependence and drug addiction (Nestler, 2004). However, the key intracellular signaling molecules that participate in regulating the alterations in gene expression induced by chronic opiate exposure remain unclear.

Recent studies (Dreyer, 2010; Hollander et al., 2010), mainly based on cocaine activity, have reported a role for miRNAs in drug addiction. This opens the door to possible miRNA-mediated involvement of opioids – including morphine – in the addictive process.

### Addiction

Addiction to drugs is a major public health problem, and represents a complex disorder with multigenic causes. Even when many humans are exposed to drugs of abuse, only some suffer from loss of control over drug use and compulsion for drug seeking and taking; factors that define the addictive situation. It is known that addiction is influenced by both the genetic constitution and the social and psychological environment in which the individual lives (Kendler et al., 2007). The pharmacological activation of brain rewards systems is largely responsible for producing addiction after drug use. Personality, genetic, and social factors are important, although drug effects in the CNS are thought to be the fundamental determinants of addiction. The genetic contribution to the risk for addiction is only close to 50% (Kendler et al., 2007), but the specific genes that are involved in the addictive process are almost completely unknown. Besides, the addictive phenotype can persist even after long periods of abstinence, implying that drugs induce long-lasting alterations in the brain that underlie addiction behaviors (for reviews on this topic, see Dreyer, 2010; Robison and Nestler, 2011).

### miRNAs

MicroRNAs (miRNAs) are ∼22 nucleotide (nt) non-coding RNAs that participate in gene regulation. They bind to 3′ untranslated regions (UTRs) of their mRNA targets, inhibiting the transcripts’ translation and/or destabilizing them (Valencia-Sanchez et al., 2006). Numerous studies have shown that multiple binding sites in the same 3′UTR confer much stronger regulation than single binding sites (Fang and Rajewsky, 2011). However, reporter assay experiments have suggested that miRNA targeting can also occur in coding regions (Kloosterman et al., 2004; Easow et al., 2007). Large-scale miRNA mis-expression studies also have suggested that binding in coding regions can confer regulation but are on average less effective than those in 3′UTRs (Baek et al., 2008; Selbach et al., 2008). MiRNAs have been shown to regulate the expression of many genes, including genes which function in the CNS. For example, miR-134 regulates dendritic spine morphology by controlling actin filament dynamics (Schratt et al., 2006), while miR-190 regulates neurogenic differentiation (NeuroD), a transcription factor that regulates the differentiation and maturation of neurons (Zheng et al., 2010c).

Over 3000 mature miRNAs have been identified in species ranging from plants to humans, suggesting that they have an important role in gene regulation. At present, a better understanding of miRNA biology, combined with the increasing availability of diverse sequenced genomes, have revealed many of the molecular mechanisms that underlie the evolution of miRNAs and their targets (Berezikov, 2011) Although the molecular mechanisms of miRNA activity are increasingly clear, the biological implications of miRNAs activity are not yet fully defined; functions including cell differentiation, proliferation, apoptosis, anti-viral defense, and cancer have been proposed, and, to an extent, validated.

It has recently been shown that miRNAs are highly expressed in the CNS, including the areas where opioid activity takes place: the brain and spinal cord (Dave and Khalili, 2010; He et al., 2010; Sanchez-Simon et al., 2010; Zheng et al., 2010b). Since the discovery that miRNAs are important regulators of gene expression, these molecules have been linked to biological processes such as drug addiction (He et al., 2010; Zheng et al., 2010a), pain perception (Kusuda et al., 2011), neuron development (Gao, 2010), viral infection (Dave and Khalili, 2010; Wang et al., 2011), and opioid receptor regulation (Wu et al., 2008; Sanchez-Simon et al., 2010).

Recent experimental work demonstrates that opioids modify the expression profile of certain mRNAs in the CNS (Wu et al., 2008; He et al., 2010; Sanchez-Simon et al., 2010). Also, recent studies, including our own results, have implicated miRNAs in addiction behaviors. Most importantly, miRNAs whose expression is altered by opioids have been shown to regulate the expression of many proteins involved in the addiction pathway (Li and van der Vaart, 2011).

To understand the biological roles of miRNAs, it is essential to identify their targets. Because only a few bases of complementarity are required between miRNAs and their target sequences, mRNA targets can often be difficult to identify, computationally or experimentally. The classical model for specific miRNA target recognition by most algorithms mainly depends on (a) the detection of seed matches and (b) the thermodynamic stability of miRNA: mRNA duplexes. Different algorithms usually produce divergent results (Ambros, 2004; Bentwich, 2005; Rajewsky, 2006; Baek et al., 2008). As miRNA recognition elements are typically found in the 3′ UTR of the target gene mRNA, bioinformatics alone can identify putative targets using resources such as miRGen database. The number of putative targets for any one miRNA has increased in recent years, making the interpretation of miRNA activity more complex. A question to be asked at present is, whether there is a relationship between the different targets a miRNA binds to, and if so, what is the meaning of this situation. Further studies are need in this field in order to elucidate the meaning of the fact that a certain miRNA has different, but perhaps functionally related, target mRNAs. Improved software programs are now able to predict the targets of miRNAs in a more efficient manner, facilitating the elucidation of miRNA function. Bioinformatic predictions (Targetscan) suggest that miRNAs target at least 60% of mammalian RNAs with conserved miRNA targets (Friedman et al., 2009).

## Opioids and miRNAs

### miR-let-7

He et al. (2010) identified a let-7 binding site in the 3′-UTR of the MOR mRNA and found that let-7 thereby represses MOR expression. They also found that morphine significantly upregulates let-7 expression in SH-SY5Y cells and in a mouse model of opioid tolerance. Inhibition of let-7 decreased brain let-7 levels and partially attenuated opioid antinociceptive tolerance in mice. Although chronic morphine treatment did not change overall MOR transcript levels, association of polysomes with MOR mRNA declined in a let-7 -dependent manner. The miRNA let-7 works as a mediator moving MOR mRNA to P-bodies, leading to translation repression. These results suggest that let-7 plays an integral role in opioid tolerance.

### miR-23b

The expression of MOR can be regulated at both the transcriptional and post-transcriptional levels. Long-term morphine treatment does not alter MOR mRNA levels (Brodsky et al., 1995), suggesting that morphine itself has no important role in the transcription of the MOR gene. Nevertheless, it has not yet been elucidated whether morphine can regulate MOR mRNA at the post-transcriptional level, by producing an interaction between *trans*-acting factors and its 3′-UTR.

Wu et al., 2008, identified miR-23b as a *trans*-acting factor that represses MOR translation efficiency through an interaction with the K box motif in the 3′-UTR of MOR1. This interaction suppresses receptor translation by inhibiting polysome-mRNA association. Later, the same group demonstrated that long-term morphine treatment increases miR-23b expression in a dose- and time-dependent manner (Wu et al., 2009). Using a translational luciferase reporter assay, these authors observed morphine-dependent suppression of reporter activity through the MOR1 3′-UTR. This finding suggests a link between MOR expression and morphine treatment at the post-transcriptional level involving miR-23b.

### miR-190

Zheng et al. (2010b) have shown that fentanyl, but not morphine, increases levels of one of the targets of miR-190, NeuroD. This group also showed that by regulating NeuroD activity, mu opioid receptor agonists modulate the stability of dendritic spines. This work is discussed elsewhere in this chapter.

### Morphine, miRNAs, and the immune system

People addicted to opioids have a higher incidence of infectious diseases, and opioids exert a profound influence in immunomodulatory activity (Nair and Schwartz, 1997). In order to understand the relationship between morphine, miRNAs, and the immune system, the following features of morphine should be considered: morphine inhibits specific immunocyte activities, such as monocyte respiratory burst (Peterson et al., 1987), chemotaxis (Stefano et al., 1996), and phagocytosis (Rojavin et al., 1993). In addition, morphine induces apoptosis of macrophages and microglia (Hu et al., 2001), decreases the levels of IFN-γ and interleukin-2 in human T cells (Nyland et al., 2003), induces the expression of HIV entry coreceptors in the immune cells, and facilitates HIV replication *in vitro* (Guo et al., 2002; Li et al., 2002; Persson et al., 2003; Steele et al., 2003).

Wang et al. (2011), showed that morphine treatment in monocytes leads to a decrease in several anti-HIV miRNAs (miR-28, 125b, 150, and 382). Interestingly, these same miRNAs were correlated with the susceptibility of monocytes to HIV-1 infection. This morphine-driven decrease in anti-HIV miRNAs disappears when antagonists of the opioid receptors are used, indicating that morphine functions through its own receptors. On the other hand, type I interferon IFN-α/β, in monocytes could induce the expression of these same anti-HIV miRNAs. Other studies have also shown that type I IFNs modulate miRNA expression in several cell systems (O’Connell et al., 2007; Pedersen et al., 2007; Ohno et al., 2009), functioning as the potent inducer of miRNAs. However, morphine co-treatment with IFN-α/β in monocytes inhibited the induction of IFN-mediated anti-HIV miRNAs (Wang et al., 2011).

HIV-1 infected opiate abusers have potential to destabilize neuronal functions, and often exhibit HIV-1 associated dementia (Bell et al., 2006; Fitting et al., 2010).

Dave and Khalili, 2010 reported that in human monocyte-derived macrophages treated with morphine, miR-15b expression levels were greatly increased. Fibroblast growth factor-2 (FGF-2), identified as a miR-15b target gene, was decreased at the protein expression levels in response to morphine. Another miRNA, miR-181b, decreased its expression levels under the same conditions. Later studies have shown that morphine induces inflammation and oxidative stress in immune cells through regulating the miR-15b and 181b, thereby contributing to expansion of the HIV-1 CNS reservoir and hence to AIDS progression.

## miR-133b

miR-133 was first characterized in mice (Lagos-Quintana et al., 2002), after which homologs were characterized in several other species including invertebrates. Each species frequently encodes multiple miRNAs with identical or similar mature sequences. Three different miR-133 sequences are known: miR-133a-1, miR-133b-2, and miR-133b.

A good example of the importance of miR-133b is represented by the work of Yu et al. (2011b). They studied the function of miR-133b during zebrafish spinal cord regeneration and showed upregulation of miR-133b expression in regenerating neurons of the brainstem after transection of the spinal cord. Inhibition of miR-133b expression by antisense morpholino (MO) application resulted in impaired locomotor recovery and reduced regeneration of axons from neurons in the nucleus of the medial longitudinal fascicle, superior reticular formation, and intermediate reticular formation. They found that miR-133b targets the small GTPase RhoA, which is an inhibitor of axonal growth, as well as other neurite outgrowth-related molecules. These results indicate that miR-133b is an important determinant in spinal cord regeneration of adult zebrafish through a reduction in RhoA protein levels by direct interaction with RhoA mRNA. These authors showed that the ability of miR-133b to suppress molecules that inhibit axon regrowth may underlie the capacity for adult zebrafish to recover locomotor function after spinal cord injury (SCI).

MicroRNAs-133b plays an important role in several regulatory processes. For example, in cardiomyocytes, miR-133b serves an antiapoptotic role by inhibiting caspase-9 (Xu et al., 2007). Among its multiple targets, miR-133b down-regulates RhoA protein expression (Care et al., 2007; Chiba et al., 2009). RhoA is strongly upregulated following SCI (Conrad et al., 2005; Erschbamer et al., 2005), and inhibition of RhoA enhances regrowth of the corticospinal tract and promotes neuroprotection by decreasing the tissue damage and cavity formation that develop after SCI (Dergham et al., 2002; Fournier et al., 2003; Tanaka et al., 2004; Hoffmann et al., 2008; Holtje et al., 2009). Considering that multiple cellular and molecular pathways are regulated by miRNAs, and that the targets of miR-133b are conserved throughout development in different species from zebrafish to mammals, it could be considered that these results (such as Yu et al., 2011b) may guide the development of novel strategies for improving functional recovery after SCI in humans. The extent to which miR-133b is involved in multiple pathological phenotypes is outstanding and highly noteworthy. Table 1 represents a summary of the involvement of miR-133b in relation to different pathological situations. Recent reports show that some miRNAs control major cancer-related signaling molecules, such as epidermal growth factor (Erkan et al., 2011), members of the p53 family (Inui et al., 2010; Ory and Ellisen, 2011), and the retinoblastoma protein (Noonan et al., 2010). In cancer, miRNAs can be divided in two separate classes: those that are tumor suppressive and those that are oncogenic. MiR-133b can participate in both systems, depending whether it is overexpressed (act as oncogenes, repressing tumor suppressor genes), or underexpressed (functioning as a tumor suppressor, negatively regulating oncogenes). Table 1 summarizes recent research regarding the role of miR-133b in multiple pathologies, including cancers. Table 2 lists cancers in which miR-133b exerts direct regulation.

**Table 1:** **Involvement of miR-133b in different physiological situations**.

Situation	Reference
Translational regulation of utrophin: miR-133b, related to Duchenne muscular dystrophy, mediates the repression, and confirms repression of miR-206	Basu et al. (2011)
Formation of homologue clusters with miR-206: dysregulation role	Nohata et al. (2012)
Upregulation during late stages of human, fetal muscle development	Koutsoulidou et al. (2011)
When downregulated, miR-133b may have important implications in pathogenesis of essential hypertension	Yu et al. (2011a)
Co-regulation of miR-133b with miR-206, novel biomarkers of Th 17-type immune reactions	Haas et al. (2011)
Desregulation of miR-133b is associated with overall survival and metastasis in colorectal cancer	Akcakaya et al. (2011)
Increase of miR-133b in mouse pectoralis muscle: regulation by myostatin	Rachagani et al. (2010)
Upregulated miR-133b in mouse liver by tyrosine hormone	Dong et al. (2010)
MiR-133b is upregulated on head and neck cancer	Liu et al. (2009)
Mir-133b is regulated by endurance exercise in human skeletal muscle	Nielsen et al. (2010)
Mir-133b is a biomarker of myocardial infection	D’Alessandra et al. (2010)
MiR-133b targets prosurvival molecules MCL-1 and BCL262 in lung cancer	Crawford et al. (2009)

**Table 2:** **Downregulation of miR-133b in different cancers**.

Type of cancer	Reference
Colorectal cancer	Suzuki et al. (2011); Hu et al. (2010); Bandres et al. (2006); Sarver et al. (2009)
Bladder cancer	Song et al. (2010)
Gastric cancer	Wu et al. (2011a)
Lung cancer	Nasser et al. (2008); Crawford et al. (2009); Wu et al. (2011b)
Esophageal squamous cell carcinoma	Kano et al. (2010)
Tongue squamous cell carcinoma	Wong et al. (2008)
Head and neck squamous cell carcinoma	Nohata et al. (2011)

The relationship of miR-133b with cancer is important to the topic of this chapter, since morphine and the synthetic compound fentanyl are widely used as the analgesic solution for long-term pain suffering in cancer patients, and it is well known that these drugs produce addiction after long-term use. MiR-133b can act as an oncogene or as a tumor suppressor, making the interaction between this miRNA and morphine crucial in the control of a defined cancer pathology. Morphine, not only because it produces addiction, but also because of its own oncogenic effect, can be considered a negative analgesic tool in some cancer patients. Future research is needed to elucidate how to overcome this possibility.

## Zebrafish as a Model to Study the Relationship between miR-133b and Morphine

Although great efforts have been made on the study of the different mechanisms that are activated by the opioid system in mammalian models, many issues regarding opioid regulation remain unknown. The zebrafish (*Danio rerio*) has been used as an experimental model, not only to study genetics and development, but also to study disease-related pathways, given its easy *in vivo* manipulation. In this sense, the zebrafish can be an important tool to analyze *in vivo* the molecular mechanisms related to the activity and function of the opioid system that cannot be fully established in other models. For instance, in contrast to mammalian embryos, which develop in the uterus and are influenced by the maternal biochemical processes, zebrafish embryos develop externally, avoiding the maternal effect on these embryos. This is essential when dealing with drug exposure, as the effects observed in mammalian embryos might be due to the susceptibility of the mother and not the embryo *per se*. The study of the morphine direct effects in the embryos will provide a better understanding on the molecular mechanisms that underlie the physical and neurobehavioral defects shown in fetuses and offspring after maternal morphine consumption (Nasiraei-Moghadam et al., 2010). Also, the endogenous opioid system has been characterized in the zebrafish, and contains a mu opioid receptor (zfMOR), two DOR duplicates (zfDOR1 and zfDOR2), a kappa opioid receptor (zfKOR) and an opioid receptor like (zfORL) gene (Barrallo et al., 2000; Rodriguez et al., 2000; Alvarez et al., 2006; Pinal-Seoane et al., 2006). Hence, the presence and the existing extensive characterization of opioid receptors in zebrafish allow us to extrapolate key components of the opioid system to other biological models.

## Opioids and the Dopaminergic System

The opioid-addiction pathway has been suggested to involve the midbrain dopaminergic neurons located within ventral tegmental areas and the nucleus accumbens (NAc). The alteration of dopamine levels in this region can produce neuronal sensitization or desensitization, depending on the drug used. It has also been established that morphine increases dopamine level through the mu opioid receptor in the NAc, which may mediate reinforcing effects of morphine (Gianoulakis, 2009). Relevant to these observations, endogenous opioid peptides, such as enkephalins or dynorphins, are upregulated in the NAc after exposure to morphine, and modulate dopamine release in the midbrain (Gieryk et al., 2010).

Hence, studies on the probable opioid regulation of dopaminergic activities in zebrafish could provide insights on mammalian embryonic development during chronic exposure to drugs.

## miR-133b and the Dopaminergic System

**Figure 1:**
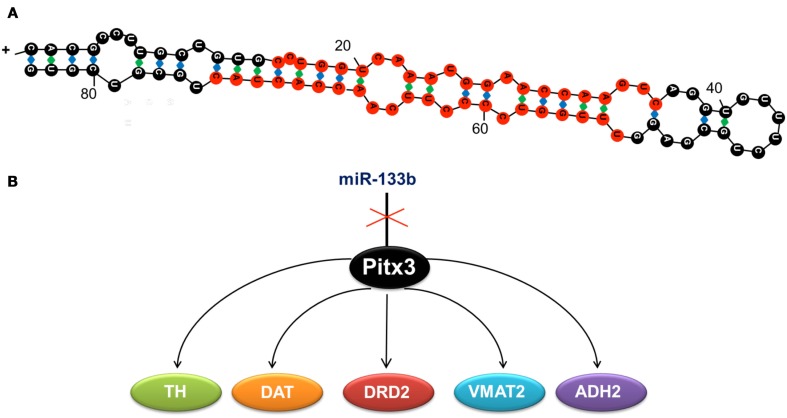
**(A)** Duplex sequence of miR-133b, formed by 84 ribonucleotides. Mature miRNA is shown in red. **(B)** miR-133b inhibits the expression of transcription factor Pitx3, whose function is to activate the expression of tyrosine hydroxylase (TH), the dopamine transporter (DAT), the dopaminergic receptor (DRD2), the monoamine vesicular transporter type 2 (VMAT2), and the aldehyde deshydrogenase 2 (ADH2). These genes determine the neuronal differentiation to the dopaminergic phenotype, so that when miR-133b is expressed, the expression of the other genes is inhibited and hence, dopaminergic differentiation is blocked.

miR-133b, regulates the differentiation, maturation, and function of dopaminergic neurons by downregulating the transcription of its target in the dopaminergic system, the homeobox gene pitx3 (Hebert and De Strooper, 2009). Pitx3 activates the transcription of genes directly involved in the differentiation of dopaminergic neurons (Figure 1), such as the tyrosine hydroxylase (*th)* and the dopamine transporter (*dat*; Kim et al., 2007).

Taking the above into consideration, we analyzed the effect of morphine on the miR-133b regulatory pathway using zebrafish embryos as a model building on well-established precedents for using zebrafish to study the role of miRs in development (Schier and Giraldez, 2006). At 24 h post fertilization (hpf), the dopaminergic system begins its differentiation and the first TH- positive neurons begin to be detected at this particular developmental stage (Filippi et al., 2007). Our previous studies also indicated that at 24 hfp, the expression of zfMOR, the putative target of morphine (de Velasco et al., 2009) is increased. Therefore, the use of 24 hpf zebrafish embryos provided information on the implication of the opioid system in the maturation and differentiation of dopaminergic neurons compared to any other stages of development, and also, demonstrated that the mu opioid receptor is functional in zebrafish and has a specific role in the development of the CNS and represents a possible pathway that leads to addiction.

## Morphine Modulates the Expression of miR-133b

By means of a miRNA array, we observed a decrease in the expression of several miRNAs after embryonic exposure to morphine at three developmental stages: 16, 24, and 48 hpf. Considering the pathways in which each miRNA could be involved, we focused on miR-133b due to its reported effect on dopaminergic neurons, an essential component in drug addiction processes. Our studies were carried out in the 24 hpf embryos, when differentiation of the zebrafish CNS begins.

After validation by qPCR of our microarray results, we observed that miR-133b level was decreased in 24 hpf embryos exposed to morphine, at two different morphine concentrations; 10 and 1 nM. The antagonist naloxone did not significantly change the expression of this miR, but it could block the morphine effect. Although more selective agonists such as DAMGO or antagonists such as CTOP were not used to define the receptor involved due to the lack of affinity of such ligands for zfMOR (de Velasco et al., 2009), the effect of morphine on miR-133b level was probably mediated by the activation of zfMOR.

## Morphine Modulates the Expression of miR-133b Target Pitx3

The transcription factor Pitx3 is a known miR-133b target. Pitx3 has been regulates the transcription of *th* and *dat*. Since miRNAs normally regulate the stability or the translation of the transcripts, by reducing miR-133b morphine should either increase the levels or the activities of these transcripts. Treatment of zebrafish embryos with 1 and 10 nM morphine increases the mRNA levels of *pitx3* and *dat* while morphine treatment decreases miR-133b level. Addition of naloxone effectively abolished the morphine-induced changes in the expression levels of miRNA-133b, *pitx3*, *th*, and *dat*, suggesting that morphine regulates the level of the dopaminergic genes via the control of miR-133b by activating zfMOR.

Although treatment of embryos with morphine clearly decreases the miR-133b level and increases Pitx3 and its targets TH and DAT levels, whether miR-133b indeed interacts with Pitx3 thereby destabilizing the transcript has not been demonstrated in zebrafish.

## The Role of zfMOR in Morphine-Induced Regulation of miR-133b Pathway

The effects of morphine on embryos are probably mediated by zfMOR, the opioid receptor that exhibits highest affinity toward morphine (de Velasco et al., 2009). In order to establish the role of zfMOR in regulating miR-133b without the availability of a zfMOR selective antagonist, we silenced (knocked down) zfMOR by morpholino oligonucleotide injection. The efficiency of the morpholino oligonucleotide to decrease the zfMOR level was determined with qRT-PCR. Injection of 0.2 μM of the morpholino oligonucleotide per embryo reduced the zfMOR transcription level by 95% (the injection of ZfMOR decreased the expression of both ZfDOR1 and ZfDOR2 by ∼2.5%, which is not statistically significant, showing the specificity of the zfMOR morpholino).

The amount of miR-133b increases within embryos when zfMOR is absent. Such an increase was not observed after the injection of a control morpholino. Furthermore, 1 or 10 nM morphine exposure did not alter the miR-133b level in embryos injected with zfMOR morpholino, while the same concentrations of morphine treatment resulted in a decrease of miR-133b levels in embryos injected with control morpholino. The increased expression in miR-133b detected in the zfMOR knock down embryos also led to a decrease of the subsequent miR-133b targets, i.e., Pitx3, TH, and DAT. Clearly, the morpholino and the opioid antagonist naloxone studies indicate zfMOR is the mediator for the morphine-induced regulation of miR-133b and its targets.

## Morphine-Induced Regulation of the miR-133b Pathway Depends on ERK1/2 Activity

**Figure 2:**
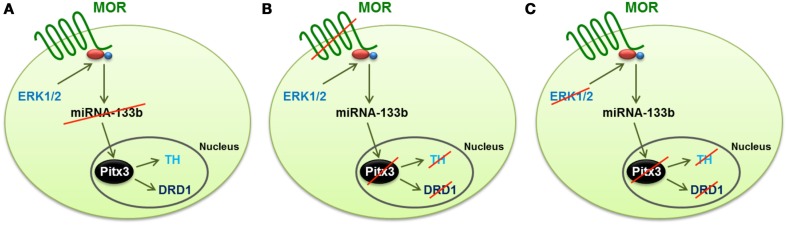
**Schematic representation of the mechanism by which morphine regulates the differentiation of dopaminergic neurons through the control of the miR-133b expression and the transcription of the genes regulated by this miRNA (Pitx3, TH, and DAT)**. **(A)** Activation of ERK1/2 signaling by MOR reduces the expression of miR-133b, and hence increases the transcription level of its target, Pitx3. This transcription factor enhances the expression of TH and DAT. **(B)** Silencing MOR by specific morpholinos produces an increase in the expression of miRNA-133b, and reduces the transcription level of Pitx3, TH, and DAT. **(C)** Inhibition of ERK1/2, even when the MOR receptor is activated by morphine, produces the same effect as knockdown of the receptor, i.e., the expression of miR-133b increases, and hence, the expression of Pitx3, TH, and DAT decreases, which reduces the level of dopaminergic neuron differentiation.

Morphine, regulates multiple signaling pathways via the mammalian receptor MOR. In the rat hippocampus, morphine activates ERK1/2 and decreases the expression level of miR-190 (Zheng et al., 2010b). Whether similar signaling mechanisms are involved in morphine-induced regulation of the miR-133b pathway in zebrafish is unknown. In order to address this possibility, we used several MAPK inhibitors, such as JNK inhibitor II, SB203580 for p38 and PD98059 and U0126 for ERK1/2, to identify the signals involved in morphine-induced miR-133a regulation. The inhibition of JNK and p38 produced a significant decrease in the miR-133b level, and hence, an increase in the level of Pitx3, TH, and DAT. In contrast, the inhibition of MEK1/2 by either U0126 or PD98059 enhanced miR-133b expression, and as a consequence, it decreased the level of Pitx3, TH, and DAT transcripts (Figure 2).

Parallel treatment of embryos with morphine in the presence of either JNK or p38 inhibitor did not eliminate the morphine-induced decrease in the miR-133b level. Probably, by activating zfMOR, morphine, via the ERK1/2 pathway, regulates the miR-133b level in the zebrafish embryos.

### Morphine regulates miR-133b expression in hippocampal neurons

When using zebrafish as a research model, there is always the question of whether a mammalian counterpart for the observed results exists. In order to determine whether the observed regulation of miR-133b by zfMOR in the zebrafish embryos has any mammalian counterparts, hippocampal neurons obtained from P1 rats were treated with 100 nM morphine. Similar to previously reported studies using mature hippocampal neuron cultures from mice chronically treated with morphine, in which miRNA array and qRT-PCR studies did not reveal any effect on the miR-133b level (Persson et al., 2003), our current studies with mature, differentiated neurons (3-week culture) revealed no effect on the expression of miR-133b when treated with morphine. However, he level of miR-133b was decreased in 1-week-old neurons treated with morphine. Thus, similar to what we have observed in the zebrafish embryos, only the miR-133b level within the immature neurons was affected by morphine treatment.

## miR-133b and the Delta Opioid Receptor

Although the mu opioid receptor displays higher affinity toward morphine than the other classical opioid receptors, delta, and kappa, an interaction between the mu and the DORs has been described *in vitro*. This interaction is thought to be responsible for faster development of morphine tolerance via the mu opioid receptor (Waldhoer et al., 2004). Moreover, knock-out mice for the DOR do not develop tolerance to morphine and do not suffer the withdrawal effect once the long-term treatment with morphine is over (Fundytus et al., 1995). Despite knowledge acquired in the past decade on the mechanisms that define opioid activity, many issues concerning the mechanisms of addiction need to be established. These goals may be achieved by analyzing the different roles of the opioid receptors apart from their analgesic functions, such as their involvement in developmental processes. For instance, the MOR and KOR increase neurogenesis (Kim et al., 2006), and the DOR acts as a neuroprotector (Narita et al., 2006). We have found that morphine protects dopaminergic neurons against glutamate-induced neurotoxicity, and this effect is mediated by the DORs (unpublished). As the mu opioid receptor regulates dopaminergic differentiation and the DORs protect these neurons, we searched for an explanation of how the DORs relate to the adverse effects of morphine.

### Role of the DORs in morphine-induced regulation of miR-133b pathway

We have studied the role of the DORs from zebrafish in the expression of miR-133b and the genes downstream in its regulatory pathway, in order to determine the specific influence of each delta receptor duplicate as a regulator of this pathway. By knocking down each DOR duplicate, both separately and simultaneously, we demonstrated that they activate the differentiation of dopaminergic neurons. Their complete absence increases miR-133b levels and therefore, decreases the mRNA levels of the genes involved in such differentiation (*pitx3*, *th*, *and dat*). However, when only one DOR is knocked down, morphine slightly decreases the expression of miR-133b, as detected in the control embryos and embryos injected with the control morpholino. These results suggest that when one DOR is not present, the other one functionally complements its role. Partial functional complementation may explain why, although some changes are observed regarding the expression of miR-133b and its related genes, these changes are not constant in untreated and morphine-treated embryos. In contrast, when both DORs are silenced, the effect produced is similar to that observed when the mu opioid receptor is knocked down, suggesting that both types of receptors, mu and delta, are involved in the differentiation of dopaminergic neurons.

## Conclusion

At present, evidence for the involvement of miRNAs in drug addiction is markedly increasing (Schaefer et al., 2007; Dreyer, 2010; Hollander et al., 2010), although these reports are mainly associated with cocaine. Aside from what we discussed in this review, there has been no direct report concerning opioid addiction.

Within the context of the dopaminergic system’s role in addictive disorders, including addiction to morphine (Flores et al., 2004; Leggio et al., 2009), we have established a pathway that may account for the observed morphine-induced increase in dopamine production (Gianoulakis, 2009). By modulating miR-133b regulatory pathways, and hence, dopaminergic differentiation, zfMOR has a specific role in the CNS and is capable of regulating transcription through miRNAs.

Our results lead us to conclude that the consequences of maternal morphine intake on the fetus could take place through the intracellular pathways of miR-133b and Pitx3. As our results suggest, neonate abstinence syndrome might be caused by the alteration in dopaminergic differentiation, induced by morphine. In addition, inhibition of ERK1/2 shows that, the closer the treatment to the timing of early CNS differentiation, the greater is the effect of this inhibition on the expression levels of the genes involved in the maturation and differentiation of dopaminergic neurons.

These data point out the importance of the developmental stage at which embryos are exposed to drugs, as exposure at different stages varies the impact of such drugs on the embryo’s development. Thus the control of miR-133b level could be a possible mechanism responsible for the development of addiction to morphine or to other drugs of abuse that increase dopamine levels in the extracellular space. These results show for the first time that the miR-133b is a possible new target for the design of new treatments against addictive disorders.

The differences in the effect of morphine on miR-133b expression of in 1-week and 3-week rat neurons demonstrate that morphine induces differentiation by decreasing the expression of this particular miRNA only in the immature neurons. Therefore, the effects of morphine consumption during pregnancy may impact neuronal differentiation, through inducing changes in miR-133b expression. These results also confirm that in mammals, morphine has the same effect as in the zebrafish in neuronal differentiation through miR-133b.

### Future perspectives

There is increasing evidence that miRNAs have a role in the control and development of many diseases: cancer (He et al., 2005; Tavazoie et al., 2008, Garzon et al., 2009), cardiovascular diseases (Zhao et al., 2005), autoimmune diseases (Sonkoly et al., 2007), neurodegenerative diseases (Fiore et al., 2008), and numerous others. In clinical practice, miRNAs can be useful as both diagnostic markers and predictors of therapeutic response (Garzon et al., 2009). Further research is warranted to elucidate the interaction between different miRNAs in order to analyze the possible therapeutic value that these post-transcriptional regulators have. The number of targets predicted for each miRNA is enormous; for example, using miR and a software, 1704 targets for miR-133b have been found. Even in this case, where we have proven that miR-133b is involved in the regulation of dopaminergic neurons, we need to find out if other physiological systems are also involved. If we can confirm different miRNAs to specifically regulate different targets, and if these miRNAs can cross-talk producing a functional result, then it is possible that therapeutic agents can be designed to rationally and specifically target the entire discovered complexity of the system. Currently, while inducing or repressing a single miRNA represents a promising therapeutic strategy for specific diseases, we can not say that every miRNA known, by itself, could be a potential therapeutic agent.

The therapeutic value of miRNAs is dictated in part by the fact that miRNAs that are upregulated in different diseases can be targeted using anti-miRNAs (antisense oligonucleotides with specific modifications; Rossbach, 2010). These microRNA inhibitors (antagomiRs) demonstrate that therapeutic targeting of miRs is possible, although these inhibitors have not yet been explored in the specific context of curing drug addiction. Besides this inhibitory therapeutic approach, indirect methods, such as downregulation of specific miRNA biogenesis pathways, could also serve as therapeutics. In the opposite scenario, when miRNAs downregulation is responsible for an abnormal function, as is the case with miRNAs downregulated in tumors, a possible therapeutic approach could be to restore mature miRNA levels in the abnormal tissue.

Accordingly, considering our results relating morphine, the mu opiod receptor, and the dopaminergic system to the miRNA miR-133b, it might be possible to design a system that could control the addiction process in which these four entities are involved, perhaps by rescuing miR-133b levels with the intention of dampening the expression of the downstream targets that positively impact dopaminergic differentiation and opioid addiction. Further research is needed to elucidate the role, and the therapeutic relevance, of miRNAs in the complexity of the addiction pathway.

## Conflict of Interest Statement

The author declares that the research was conducted in the absence of any commercial or financial relationships that could be construed as a potential conflict of interest.
